# Effects of knee position on blood loss following total knee arthroplasty: a randomized, controlled study

**DOI:** 10.1186/s13018-015-0213-9

**Published:** 2015-05-17

**Authors:** Jun Liu, Yao-min Li, Jian-gang Cao, Lei Wang

**Affiliations:** Tianjin Medical University, 30070 Tianjin, China; Center for Joint Diseases, Tianjin Hospital, 300211 Tianjin, China; Department of Rehabilitation, Tianjin Hospital, 300211 Tianjin, China

**Keywords:** Total knee arthroplasty, Blood loss, Knee position

## Abstract

**Objective:**

Blood loss following total knee arthroplasty is a serious side-effect of surgery and impacts on patient recovery and quality of life. The aim of this study was to assess the effect of postoperative knee position during recovery on blood loss and range of motion.

**Methods:**

One hundred consecutive patients, with stage III or IV degenerative osteoarthritis, were enrolled in the study and randomized equally between two treatment groups: flexion and extension. In the flexion group, the affected leg was elevated postoperatively by 45° at the hip, with 45° flexion at the knee, while patients in the extension group had the knee extended fully. Blood loss, pre- and postoperative hemoglobin levels, and range of motion were recorded together with duration of hospital stay and complications.

**Results:**

Calculated blood loss, hidden blood loss, and postoperative hemoglobin levels between the two groups were significantly different, with patients in the flexion group experiencing lower blood loss than those in the extension group (*P* < 0.05). After 6-week rehabilitation, patients from both groups attained a similar range of motion in the joint. Duration of hospital stay was shorter in the flexion group by 1.6 days. Wound infection rates were similar in both groups, and we observed no proven deep vein thrombosis.

**Conclusions:**

Postoperative elevation of the hip by 45°, with 45° knee flexion, is an effective and simple method of reducing blood loss and hospital stay following unilateral primary total knee arthroplasty.

## Introduction

With an increasing number of aged people suffering from degenerative osteoarthritis of the knee, total knee arthroplasty (TKA) has become an important method for relieving pain and improving quality of life. The procedure involves extensive soft tissue release and bone incisions, resulting in significant blood loss. The resulting reduction in hemoglobin level may have a serious impact on patient health, especially for older individuals with lower hematopoietic ability. Allogenic blood transfusions are generally used to prevent postoperative anemia. However, transfusions are associated with additional risks, such as transmission of infectious disease, transfusion-related acute lung injury, hemolytic reactions, fluid overload, increased rate of postoperative infection [1], and immunosuppression [2]. In addition, allogenic blood transfusions can also lengthen the duration of hospital stay and increase the cost of treatment.

Clinically, blood loss influences the restoration of a functional range of motion (ROM), an important factor when evaluating the success of TKA, because different ROMs are needed for different types of activity. For example, 67° of flexion are required for the swing phase of gait, 83° to climb stairs, 90° to descend stairs, and at least 93° to rise from a chair. Recovery of a good range of motion is vital for an optimal result, and small changes in maximum flexion can have profound effects on functional capability [3]. The greatest improvement in flexion has been found at the time of patients discharged and 6 weeks postoperatively. Postoperative knee swelling, mainly due to hidden hemorrhage around the knee joint, impedes early rehabilitation. Therefore, minimizing blood loss following surgery has a positive impact on both clinical recovery and in reducing the need for transfusion, avoiding the associated risks [4].

Various procedures have been used to reduce blood loss associated with TKA, including tourniquet use during surgery [5], drain placement protocols [6], bipolar sealers [7], autologous platelet gels [8], and the most important method is using tranexamic acid by intravenous or intra-articular injections [9]. Postoperative limb positioning has also been investigated in several studies but has shown conflicting results [5, 10–15]. A systematic review of these studies concluded that a 48–72-h postoperative knee flexion protocol should be implemented as a simple and inexpensive method of reducing blood loss and increasing ROM following TKA [16].

There is no definitive consensus upon whether it is exact limb position or duration of flexion which contributes most to the benefits seen with postoperative flexion protocols. The aim of the present study was therefore to examine the effect of knee position on blood loss and related parameters at 48 h following total knee arthroplasty, during a randomized, prospective comparative study.

## Materials and methods

### Study design

This study was approved by the ethics committee of hospital [Tianjin Hospital], and we obtained informed consent from all patients, after explaining the benefits and risks of the study. We conducted a prospective, randomized clinical study, between June 2013 and June 2014, including patients diagnosed with stage III or IV degenerative osteoarthritis of the knee. Diagnosis was based on medical history, clinical examination, and X-ray of the standard weight-bearing knee. Patients were excluded based on the following criteria: history of knee trauma or knee surgery, inflammatory polyarthritis (*e.g.*, rheumatoid arthritis), neuromuscular diseases, disorders of the hips, metabolic bone disease, and other serious medical conditions (for example, neoplasms, chronic atrial fibrillation, or diabetes mellitus).

One hundred patients were enrolled in the study. An equal number of patients were randomly allocated to either flexion or extension groups, using a random number list generated at the end of surgery. In the flexion group, the affected leg was elevated 45° at the hip using an inactive continuous passive motion machine with 45° of flexion at the knee, while patients in the extension group had the knee extended fully. The two groups were well matched for age, gender, affected side, preoperative hemoglobin (Hb) levels, and ROM of the knee. Patient demographics and baseline measurements are summarized in Table 1. Aspirin and other agents affecting clotting function were terminated prior to surgery for 7 days.

**Table 1:** Demographics and baseline measurements

	Flexion group	Extension group	*P* value
Age (years)	73.1 ± 5.1	72.4 ± 4.6	0.68
Gender (female/male)	34/16	32/18	0.67
Affected side (L/R)	22/28	24/26	0.69
Preoperative Hb (g/dL)	13.3 ± 1.4	12.8 ± 1.7	0.34
Preoperative ROM	93.9 ± 18.9	92.3 ± 21.5	0.80

*L* left, *R* right, *Hb* hemoglobin, *ROM* range of motion

### Surgical procedure

All operations were performed under spino-epidural anesthesia by the same group of surgeons skilled in TKA. Tourniquets were not used during the procedure. All surgery was performed via a medial parapatellar approach, incising the quadriceps tendon along the medial border. The synovial capsular reflection and patellar tendon fat pad were excised. Osteophyte excision and soft tissue release were performed where necessary. The implant used was a posterior cruciate ligament-substituting, total knee prosthetic component (GENESIS II, Smith & Nephew Inc., Memphis, TN, USA). The patella was reshaped to better match the shape of the femoral component trochlea. To reduce blood loss from the femoral hole, an intramedullary plug with bone grafts was used prior to wound closure. A high-elasticity bandage was applied to compress the wound. No drain was placed.

### Rehabilitation program

All patients received standard thromboprophylaxis in the form of enoxaparin (40 mg), with the first dose administered 12-h preoperatively and continued throughout hospital stay. If required, a patient-controlled analgesia system machine was provided for up to 48 h postoperatively, and thereafter, NSAID-based analgesia was used to control postoperative pain. Wound dressings were changed at 48 h, and the bandage was not wrapped so tightly as to hinder blood circulation in the extremities. Active isometric quadriceps, initiative straight leg raises, and extension–flexion motion were encouraged 48 h postoperatively, and ambulation with partial weight bearing was permitted under the supervision of a physical therapist. During the later part of hospital stay, the affected leg would always be placed back in the flexion or extension position after physical therapy. The transfusion trigger was defined as a hemoglobin level of less than 8 g/dl at 48 h postsurgery, with additional anemia symptoms, such as tachycardia and dizziness, measured.

### Follow-up assessment

Patients were assessed by a surgeon blinded to the postoperative limb position grouping, with affected legs left in extension at the time of examination. Calculated total blood loss (CBL) was obtained using the formula reported by Gross [17]. Hb levels were measured preoperatively and at 48 h postoperatively. Hidden blood loss (HBL) was calculated by subtracting intraoperative blood loss and postoperative wound blood loss (obtained by weighing soiled dressings) from the CBL at the 48th postoperative hour. The ROM was measured preoperatively and at 6 weeks postoperatively. The length of hospital stay was recorded as an economic factor. All other complications were recorded, including wound infection and deep vein thrombosis (DVT). Any clinical suspicion of DVT was investigated by Doppler ultrasound.

### Statistical analysis

Statistical analysis was performed using SPSS for Windows 11.5 software (SPSS, Chicago, Illinois). Continuous data were expressed as means (± standard deviation). A two-tailed Student’s *t* test was used to compare continuous variables, while chi-square test was used for nominal data. The level of significance was set at *P* < 0.05.

## Results

Study outcomes are summarized in Table 2. We observed a significant difference in CBL, HBL, and postoperative Hb levels between the two groups (*P* < 0.05). Based on transfusion criteria, three patients in the flexion group and nine patients in the extension group required transfusions, although this difference was not statistically significant. After 6 weeks of rehabilitation, both groups had a similar ROM without statistical difference and overall patients in the flexion group spent less time in hospital than those in the extension group. The most common postoperative complications were wound infections: three superficial infections occurred in the flexion group and two in the extension group, and two deep infections occurred in the flexion and one in the extension groups. There were no cases of DVT observed in this study.

**Table 2:** Clinical outcome and complications

	Flexion group	Extension group	*P* value
CBL (ml)	1008.4 ± 102.6	1212.0 ± 113.9	0.00
HBL (ml)	505.1 ± 28.0	617.5 ± 52.4	0.00
Postoperative Hb (g/dL)	10.8 ± 1.1	10.0 ± 1.3	0.04
Transfusion	3	9	0.06
ROM	105.8 ± 11.6	104.5 ± 9.2	0.70
Duration	10.1 ± 1.1	11.7 ± 1.3	0.00
Complications			0.46
Superficial/deep infection	3/2	2/1	
DVT	0	0	

*CBL* calculated total blood loss, *HBL* hidden blood loss, *Hb* hemoglobin, *ROM* range of motion, *DVT* deep vein thrombosis

## Discussion

Blood loss following TKA is thought to be one of the major contributing factors to a better functional outcome. Many methods have therefore been employed to decrease blood loss and aid good postoperative ROM. The aim of the present study was to examine the effect of adopting a postoperative knee flexion position after TKA: our results showed that this position had a positive impact on surgery outcome. Using a 45° hip elevation and 45° knee flexion position for 48 h postoperatively resulted in significantly lower blood loss than that seen in the knee extension group.

Previous studies have examined the effect of a greater angle of hip elevation and have shown no significant difference in total blood loss and hemoglobin levels [10, 13–15]; however, this may be due to femoral and popliteal veins being so curved as to hinder venous return [5]. Patients may be more uncomfortable while on the flexion regime, due to increased immobility, increased wound stretch, and pain receptor stimulation in flexion [14]. To avoid this, we elevated the hip by 45° and put the knee into a 45° flexion position, showing similar results to the study of Li *et al.* [5]. Blood loss after wound closure comes from extensive soft tissue release in the early postoperative period, and passive motion during this time also influences blood coagulation. The greatest blood loss following TKA has previously been reported in patients who had immediate continuous passive motion following surgery [18]. One hypothesis for the reason why flexion can reduce bleeding is that vessel angulation occurs with knee flexion, resulting in increased local tension and decreased venous return. This mechanism is thought to be effective only for flexion protocols of 48 h or longer [16]. Compression was also maintained for 48 h postoperatively because patients treated by 48 h compression with high-elasticity bandages have shown quicker postoperative recovery, with shorter hospital stays and a greater range of flexion on discharge [19]. Based on these findings, all the patients’ affected legs in our study were maintained in flexion or extension for 48 h postoperatively. After this period, continuous passive motion was then encouraged. Suction drainage was not used, as it may have negated any tamponade effect from the reduction in intra-articular knee volume. Tourniquets were also not used because TKA without tourniquet shows superior early postoperative rehabilitation, fewer thromboembolic events, and other related complications [20]. Duration of hospital stay was also shorter in the flexion group by only 1.6 days. The reason why decreased length of stay was seen in the flexion group which what we speculated was that these patients had less blood loss with better general health and more confidence of physical practice. These results were in accordance with results of a previous study, in which increasing knee circumference and area of ecchymosis at 3 days was less in the flexion group [5]. In contrast, no significant difference between flexion and extension groups has been reported where clinical parameters have only been measured for a shorter period of up to 6–24 h, postoperatively [13–15].

Restoration of functional ROM also benefits from minimizing hidden blood loss during rehabilitation after TKA, for several reasons. Firstly, patients take less time to regain an adequate level of health, important for resumption of exercise. Secondly, a lower degree of joint effusion and swelling relieves the burden on the quadriceps, allowing straight leg raising exercises to be practiced. At the 6-week follow-up assessment, patients in the knee flexion group showed statistically better outcomes for ROM and straight leg raise exercises than those in the extension group in our study.

It must be borne in mind that in this study, we only conducted a short follow-up, examining early blood parameters and range of movement because we were not able to control rehabilitation regime and medical care for a longer period following discharge. Another limitation of this study is the fact that small patient numbers meant that we were unable to test a greater number of protocols, looking at differing angles and duration of flexion. Thirdly, we must be cautious when interpreting the statistical difference. The SD of postoperative Hbs for extension and flexion group (1.1 and 1.3) were greater than the average value of 0.8, which may undermine the effect of flexion knee position. Further studies are therefore required to gain consensus regarding the benefit of postoperative limb position in patients following TKA.

## Conclusion

Maintaining a position with 45° hip elevation and 45° knee flexion, for 48 h postoperatively, is an effective method for reducing early blood loss and increasing functional range of motion following TKA.
